# A novel sequence space related to $\mathcal{L}_{p}$ defined by Orlicz function with application in pattern recognition

**DOI:** 10.1186/s13660-017-1541-6

**Published:** 2017-12-06

**Authors:** Mohd Shoaib Khan, QM Danish Lohani

**Affiliations:** Department of Mathematics, South Asian University, New Delhi, 110021 India

**Keywords:** 40H05, 46A45, clustering, double sequence, *k*-means clustering, Orlicz function

## Abstract

In the field of pattern recognition, clustering groups the data into different clusters on the basis of similarity among them. Many a time, the similarity level between data points is derived through a distance measure; so, a number of clustering techniques reliant on such a measure are developed. Clustering algorithms are modified by employing an appropriate distance measure due to the high versatility of a data set. The distance measure becomes appropriate in clustering algorithm if weights assigned at the components of the distance measure are in concurrence to the problem. In this paper, we propose a new sequence space $\mathcal{{M}} ( \phi,p,\mathcal{{F}} )$ related to $\mathcal{L}_{p}$ using an Orlicz function. Many interesting properties of the sequence space $\mathcal{{M}} ( \phi,p,\mathcal{{F}} )$ are established by the help of a distance measure, which is also used to modify the *k*-means clustering algorithm. To show the efficacy of the modified *k*-means clustering algorithm over the standard *k*-means clustering algorithm, we have implemented them for two real-world data set, viz. a two-moon data set and a path-based data set (borrowed from the UCI repository). The clustering accuracy obtained by our proposed clustering algoritm outperformes the standard *k*-means clustering algorithm.

## Introduction

Clustering is the process of separating a data set into different groups (clusters) such that objects in the same cluster should be similar to one another but dissimilar in another cluster [1–3]. It is a procedure to handle unsupervised learning problems appearing in pattern recognition. The major contribution in the field of clustering came due to the pioneering work of MacQueen [1] and Bazdek [2]. The *k*-means clustering algorithm was introduced by MacQueen [1], which is based on the minimum distance of the points from the center. The variants of *k*-means clustering algorithms were proposed to solve different types of pattern recognitions problems (see [4–7]). The clustering results of *k*-means or its variant can be further enhanced by choosing an appropriate distance measure. Therefore, the distance measure has a vital role in the clustering.

**Figure 1:**
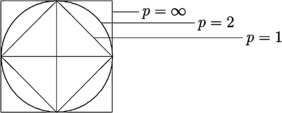
**Geometry of** $\pmb{l_{p}}$ **norm.**

Clustering process is usually carried out through the $l_{2}$ distance measure [8], but, due to its trajectory, sometimes it fails to offer good results. Suppose that two points *x* and *y* are selected on the boundary of the square (case $p = 1$) and let *z* be the center (Figure 1). Then $l_{1}$ will fail to distinguish *x* and *y*, but these points may be distinguished by $l_{2}$. If the points *x* and *y* are on the circumference of the circle, then $l_{2}$ will fail to distinguish them. Moreover, the $l_{p}$ ($p \ge 1$) distance measures are not flexible, so they cannot be modified as per the need of the clustering problem. Hence, clustering results derived through distance-dependent algorithms basically depend upon two properties of a distance measure: (1) trajectory and (2) flexibility. Till now, we have not come across to any distance measure that offers a guaranteed good result for every clustering problems. Clustering is carried out by using other variants of the $l_{p}$ distance measure. The distance measure of the sequence space $l^{p,q}$, $1 \le p,q \le \infty$, introduced by Kellogg [9] and further studied by Jovanovic and Rakocevic [10], Oscar and Carme [11], and Ivana et al. [12] offers more flexibility in comparison to $l_{p}$ due to involvement of additional parameter *q*. Sargent [13] introduced another interesting sequence spaces $m(\varphi )$ and $n(\varphi )$ closely related to $l_{p}$. Some useful extensions of $m(\varphi )$ and $n(\varphi )$ sequence spaces were proposed by Tripathy and Sen [14], Mursaleen [15, 16], and Vakeel [17]. Malkowsky et al. [18] defined a matrix mapping into the strong Cesàro sequence space [19] and studied the modulus function. Recently, for first time, Khan et al. [20] defined a distance measure of the double sequence of $\mathcal{{M}}(\phi )$ and $\mathcal{{N}}(\phi )$ to cluster the objects. Moreover, Khan et al. in [38, 39] defined some more similarity measures by using distance measures of the double sequences in the uncertain environment. Mohiuddine and Alotaibi applied measures of noncompactness to solve an infinite system of second-order differential equations in $\ell_{p}$ spaces [21, 22]. The double sequence space is further studied by Mursaleen and Mohiuddine [23], Altay and Başar [24, 25], Başar and Şever [26], and Esi and Hazarika [27]. Moreover, an Orlicz function and a fuzzy set are also used to define other types of double sequence spaces [18, 28–31]. The convergence of difference sequence spaces is discussed in [29, 32, 33].

In this paper, we define a new double sequence space $\mathcal{{M}}(\phi,p,\mathcal{{F}})$ related to $\mathcal{L}_{p}$ using the following Orlicz function:

$$\begin{aligned} &\mathcal{{M}}(\phi,p,\mathcal{{F}}) \\ &\quad = \biggl\{ x = \{ x_{mn}\} \in \Omega:\sup_{s,t \ge 1}\sup_{\zeta \in \mathcal{{U}}_{st}}\frac{1}{\phi_{st}} \sum_{m,n \in \zeta} \biggl( \mathcal{{F}} \biggl( \frac{ \vert x_{mn} \vert }{\rho} \biggr) \biggr)^{p} < \infty, \text{ for some }\rho > 0 \biggr\} . \end{aligned} $$

Obviously, $\mathcal{{M}}(\phi,p,\mathcal{{F}})$ is a norm space, and hence the induced distance measure is represented as

$$d_{M(\phi,p,\mathcal{{F}})}(x,y) = \biggl( \sup_{s,t \ge 1}\sup _{\zeta \in \mathcal{{U}}_{st}}\frac{1}{\phi_{st}}\sum_{m,n \in \zeta} \biggl( F \biggl( \frac{ \vert x_{mn} - y_{mn} \vert }{\rho} \biggr) \biggr)^{p} \biggr)^{\frac{1}{p}}. $$

The parameters *ϕ*, *p* and Orlicz function $\mathcal{{F}}$ of $\mathcal{{M}}(\phi,p,\mathcal{{F}})$ brings flexibility in the induced distance measure $d_{M(\phi,p,\mathcal{{F}})}$, which helps the user to modify it as per need of the clustering problem. Besides, defining the distance measure of $\mathcal{{M}}(\phi,p,\mathcal{{F}})$, we have also studied some of its mathematically established properties. Finally, the distance measure of $\mathcal{{M}}(\phi,p,\mathcal{{F}})$ is used in the *k*-means clustering algorithm, which clusters real-world data sets such as two-moon data set and path-based data set. The clustering results obtained by the modified clustering algorithm is compared with the *k*-means clustering algorithm to show its efficacy.

## Preliminaries

Throughout the paper, $l_{\infty}$, *c*, and $c_{0}$ denote the Banach spaces of bounded, convergent, and null sequences; *ω*, $\mathbb{N}$, and $\mathbb{R}$ denote the sets of real (ordinary or single) sequences, natural numbers, and real numbers, respectively.

### Orlicz function [34]

A function $\mathcal{{F}}:[0,\infty ) \to [0,\infty )$ is called an Orlicz function if

(i) $\mathcal{{F}}(0) = 0$, $\mathcal{{F}}(x) > 0$ for $x > 0$, and $\mathcal{{F}}(x) \to \infty$ as $x \to \infty$;
(ii) $\mathcal{{F}}$ is convex;
(iii) $\mathcal{{F}}$ is nondecreasing; and
(iv) $\mathcal{{F}}$ is continuous from the right of 0.

An Orlicz function $\mathcal{{F}}$ is said to satisfy $\Delta_{2}$-condition for all values of *x* if there exists a constant $K > 0$ such that $\mathcal{{F}}(2x) \le K\mathcal{{F}}(x)$ for all $x \ge 0$. The $\Delta_{2}$-condition is equivalent to $\mathcal{{F}}(Lx) \le K\mathcal{{F}}(x)$ for all values of $x > 0$ and for $L > 1$. An Orlicz function $\mathcal{{F}}$ can always be represented in the following integral form:

$$\mathcal{{F}}(x) = \int_{0}^{x} \eta (t)\,dt, $$

where *η*, known as the kernel of $\mathcal{{F}}$, is right-differentiable for $t \ge 0$, $\eta (0) = 0$, $\eta (t) > 0$ for $t > 0$, *η* is nondecreasing, and $\eta (t) \to \infty$ as $t \to \infty$.

Let $\mathcal{{C}}$ be the space of finite sets of distinct positive integers. Given any element *σ* of $\mathcal{{C}}$. Let $c(\sigma )$ be the sequence $\{ c_{n}(\sigma )\}$ such that $c_{n}(\sigma ) = 1$ if $n \in \sigma$ and $c_{n}(\sigma ) = 0$ otherwise. Further, let

$$\mathcal{{C}}_{s} = \Biggl\{ \sigma \in \mathcal{{C}}:\sum _{n = 1}^{\infty} c_{n}(\sigma ) \le s \Biggr\} \quad (\mbox{cf. [6]}) $$

be the set of those *σ* whose support has cardinality at most *s*, and

$$\Phi = \biggl\{ \phi = \{ \phi_{n}\} \in \omega:\phi_{1} > 0,\Delta \phi_{n} \ge 0\mbox{ and }\Delta \biggl( \frac{\phi_{n}}{n} \biggr) \le 0\ (n = 1,2, \ldots ) \biggr\} , $$

where $\Delta \varphi_{n} = \varphi_{n} - \varphi_{n - 1}$.

For $\varphi \in \Phi$, the sequence space, introduced by Sargent [13] and known as Sargent’s sequence space, is defined as follows:

$$m(\phi ) = \biggl\{ x = \{ x_{n}\} \in \omega:\sup _{s \ge 1}\sup_{\sigma \in C_{s}} \biggl( \frac{1}{\phi_{s}}\sum _{n \in \sigma} \vert x_{n} \vert \biggr) < \infty \biggr\} . $$

Let Ω be the set of all real-valued double sequences, which is a vector space with coordinatewise addition and scalar multiplication. A double sequence $x = \{ x_{mn}\}$ of real numbers is said to be *bounded* if $\Vert x \Vert _{\infty} = \sup_{m,n} \vert x_{mn} \vert < \infty$. We denote the space of all bounded double sequences by $\mathcal{L}_{\infty} $. Consider a sequence $x = \{ x_{mn}\} \in \Omega$. If for every $\varepsilon > 0$, there exist $n_{ \circ} = n_{ \circ} (\varepsilon ) \in \mathbb{N}$ and $\ell \in \mathbb{R}$ such that

$$\vert x_{mn} - \ell \vert < \varepsilon $$

for all $m,n > n_{ \circ} $ then we say that the double sequence *x* is *convergent* in the *Pringheim sense* to the limit *ℓ* and write $\mathcal{{P}} \mbox{-} \lim x_{mn} = \ell$. By $\mathcal{{C}}_{p}$ we denote the space of all convergent double sequences in the Pringsheim sense. It is well known that there are such sequences in the space $\mathcal{{C}}_{p}$ but not in the space $\mathcal{L}_{\infty} $. So, we can consider the space $\mathcal{{C}}_{bp}$ of double sequences that are both convergent in the Pringsheim sense and bounded, that is, $\mathcal{{C}}_{bp} = \mathcal{{C}}_{p} \cap \mathcal{L}_{\infty} $. A double sequence $x = \{ x_{mn}\}$ is said to *converge regularly to* *ℓ* (shortly, *r*-convergent *to* *ℓ*) if *x* is $\mathcal{{P}}$-convergent to *ℓ* and the limits $x_{m}: = \lim_{n}x_{m,n}$ ($m \in \mathbb{N}$) and $x_{n}: = \lim_{m}x_{m,n}$ ($n \in \mathbb{N}$) exist. Note that, in this case, the limits $\lim_{m}\lim_{n}x_{m,n}$ and $\lim_{n}\lim_{m}x_{m,n}$ exist and are equal to the $\mathcal{{P}}$-limit of *x*. Therefore, *ℓ* is called the *r*-limit of *x*.

In general, for any notion of convergence *ν*, the space of all *ν*-convergent double sequences will be denoted by $\mathcal{{C}}_{\nu} $, and the limit of a *ν*-convergent double sequence *x* by $\nu \mbox{-} \lim_{m,n}x_{mn}$, where $\nu \in \{ \mathcal{{P}},bp,r\}$.

Başar and Sever [26] have introduced the space $\mathcal{L}_{p}$ of *p*-summable double sequences corresponding to the space $l_{p}$ ($p \ge 1$) of single sequences as

$$\mathcal{L}_{p}: = \biggl( \{ x_{mn}\} \in \Omega:\sum _{m,n} \vert x_{mn} \vert ^{p} < \infty \biggr)\quad (1 \le p < \infty ) $$

and examined some properties of the space. Altay and Başar [25] have generalized the set of double sequences $\mathcal{L}_{\infty} $, $\mathcal{{C}}_{p}$, and $\mathcal{{C}}_{bp}$ etc. by defining $\mathcal{L}_{\infty} (t) = ( \{ x_{mn}\} \in \Omega:\sup_{m,n \in \mathbb{{N}}} \vert x_{mn} \vert ^{t_{mn}} < \infty )$, $\mathcal{{C}}_{p}(t) = ( \{ x_{mn}\} \in \Omega:\mathcal{{P}}\mbox{-}\lim_{m,n \to \infty} \vert x_{mn} - \ell \vert ^{t_{mn}} < \infty )$, and $\mathcal{{C}}_{bp}(t) = \mathcal{{C}}_{p} \cap \mathcal{L}_{\infty} $, respectively, where $t = \{ t_{mn}\}$ is a sequence of strictly positive reals $t_{mn}$. In the case $t_{mn} = 1$ for all $m,n \in \mathbb{N}$, $\mathcal{L}_{\infty} (t)$, $\mathcal{{C}}_{p}(t)$, and $\mathcal{{C}}_{bp}(t)$ reduce to the sets $\mathcal{L}_{\infty} $, $\mathcal{{C}}_{p}$ and $\mathcal{{C}}_{bp}$, respectively.

Now just to have a better idea about other convergences, especially the linear convergence, we first consider the isomorphism defined by Zelster [35] as

1 $$ \begin{gathered} T:\Omega \to \omega, \\ x \mapsto z = (z_{i}): = ( x_{\chi^{ - 1}(i)} ), \end{gathered} $$

where $\chi:\mathbb{N} \times \mathbb{N} \to \mathbb{N}$ is the bijection defined by

$$\begin{gathered} \chi \bigl[(1,1) \bigr] = 1,\qquad \chi \bigl[(1,2) \bigr] = 2, \qquad \chi \bigl[(2,2) \bigr] = 3,\qquad \chi \bigl[(2,1) \bigr] = 4 \\ \vdots \\ \chi \bigl[(1,n) \bigr] = (n - 1)^{2} + 1,\qquad \chi \bigl[(2,n) \bigr] = (n - 1)^{2} + 2,\qquad \ldots, \\ \chi \bigl[(n,n) \bigr] = (n - 1)^{2} + n,\qquad \chi \bigl[(n,n - 1) \bigr] = n^{2} - n + 2,\qquad \ldots,\qquad \chi \bigl[(n,1) \bigr] = n^{2}, \\ \vdots \end{gathered} $$

Let us consider a double sequence $x = \{ x_{mn}\}$ and define the sequence $s = \{ s_{mn}\}$ via *x* by

$$s_{mn}: = \sum_{i,j}^{m,n} x_{ij}\quad (m,n \in \mathbb{N}). $$

For brevity, here and in what follows, we abbreviate the summations $\sum_{k = 1}^{\infty} \sum_{l = 1}^{\infty} $ and $\sum_{k = 1}^{m} \sum_{l = 1}^{n}$ by $\sum_{i,j = 1}^{\infty,\infty} $ and $\sum_{i,j = 1}^{m,n}$, respectively. Then the pair $(x,s)$ and the sequence $s = \{ s_{mn}\}$ are called a double series and the sequence of partial sums of a double series, respectively. Let *λ* be the space of double sequences, converging with respect to some linear convergence rule $\mu \mbox{-} \lim:\lambda \to \mathbb{R}$. The sum of a double series $\sum_{i,j = 1}^{\infty,\infty} x_{ij}$ with respect to this rule is defined by $\mu\mbox{-} \sum_{i,j = 1}^{\infty,\infty} x_{ij}: = \mu \mbox{-} \lim s_{mn}$.

In this paper, we define an analogoue of Sargent’s sequence in the double sequence space Ω. For this, we first suppose that $\mathcal{{U}}$ is the space whose elements are finite sets of distinct elements of $\mathbb{N} \times \mathbb{N}$ obtained by $\sigma \times \varsigma$, where $\sigma \in \mathcal{{C}}_{s}$ and $\varsigma \in \mathcal{{C}}_{t}$ for each $s,t \ge 1$. Therefore any element *ζ* of $\mathcal{{U}}$ means $(j,k)$; $j \in \sigma$ & $k \in \varsigma$ having cardinality atmost *st*, where *s* is the cardinality with respect to *m*, and *t* is the cardinality with respect to *n*. Here, the product say *c* of *st* may be same for differnt sets of positive integers $k,l$, but in that case, $\mathcal{{U}}_{kl}$ is different from $\mathcal{{U}}_{st}$. Given any element *ζ* of $\mathcal{{U}}$, we denote by $c(\zeta )$ the sequence $\{ c_{mn}(\zeta )\}$ such that

$$c_{mn}(\zeta ) = \textstyle\begin{cases} 1 &\mbox{if } (m,n) \in \zeta, \\ 0 &\mbox{otherwise}. \end{cases} $$

Further, let

$$\mathcal{{U}}_{st} = \Biggl\{ \zeta \in \mathcal{{U}}:\sum _{m,n = 1}^{\infty,\infty} c_{mn}(\zeta ) \le st \Biggr\} $$

be the set of those *ζ* whose support has cardinality at most *st*, and let

$$\begin{aligned} \Theta &= \biggl\{ \phi = \{ \phi_{mn}\} \in \Omega: \phi_{11} > 0,\Delta_{10}\phi_{mn}, \Delta_{01}\phi_{mn},\Delta_{11}\phi_{mn} \ge 0\mbox{ and} \\ &\quad {} \Delta_{10} \biggl( \frac{\phi_{mn}}{mn} \biggr), \Delta_{01} \biggl( \frac{\phi_{mn}}{mn} \biggr) , \Delta_{11} \biggl( \frac{\phi_{mn}}{mn} \biggr) \le 0\ (m,n = 1,2, \ldots ) \biggr\} , \end{aligned} $$

where $\Delta_{10}\varphi_{mn} = \varphi_{mn} - \varphi_{m - 1n}$, $\Delta_{01}\varphi_{mn} = \varphi_{mn} - \varphi_{mn - 1}$, $\Delta_{11}\varphi_{mn} = \varphi_{mn} - \varphi_{m - 1n - 1}$.

For $\varphi \in \Theta$, we define the sequence space

$$M(\phi,\mathcal{{F}}) = \biggl\{ x = \{ x_{mn}\} \in \Omega:\sup _{s,t \ge 1}\sup_{\zeta \in \mathcal{{U}}_{st}}\frac{1}{\phi_{st}}\sum _{m,n \in \zeta} \mathcal{{F}} \biggl( \frac{ \vert x_{mn} \vert }{\rho} \biggr) < \infty,\mbox{ for some }\rho > 0 \biggr\} . $$

Throughout the paper, $\sum_{m,n \in \zeta} $ means $\sum_{m \in \sigma} \sum_{n \in \varsigma} $.

The spaces $\mathcal{{M}}(\phi,\mathcal{{F}})$, $\mathcal{L}_{p}$, and $\mathcal{L}_{\infty} $ can be extended to $\mathcal{{M}}(\phi,p,\mathcal{{F}})$, $\mathcal{L}_{p}(\mathcal{{F}})$, and $\mathcal{L}_{\infty} (\mathcal{{F}})$ as follows:

$$\begin{gathered} \mathcal{{M}}(\phi,p,\mathcal{{F}}) \\ \quad = \biggl\{ x = \{ x_{mn}\} \in \Omega:\sup_{s,t \ge 1}\sup _{\zeta \in \mathcal{{U}}_{st}}\frac{1}{\phi_{st}}\sum_{m,n \in \zeta} \biggl( \mathcal{{F}} \biggl( \frac{ \vert x_{mn} \vert }{\rho} \biggr) \biggr)^{p} < \infty,\mbox{ for some }\rho > 0 \biggr\} , \\ \mathcal{L}_{p}(\mathcal{{F}}) = \biggl\{ \{ x_{mn}\} \in \Omega:\sum_{m,n} \biggl( \mathcal{{F}} \biggl( \frac{ \vert x_{mn} \vert }{\rho} \biggr) \biggr)^{p} < \infty,\mbox{ for some }\rho > 0 \biggr\} \quad (1 \le p < \infty ), \\ \mathcal{L}_{\infty} (\mathcal{{F}}) = \biggl\{ \{ x_{mn}\} \in \Omega:\sup_{m,n}\mathcal{{F}} \biggl( \frac{ \vert x_{mn} \vert }{\rho} \biggr) < \infty,\mbox{ for some }\rho > 0 \biggr\} . \end{gathered} $$

Now, if we take the cardinality *t* with respect to *n* as 1, then $\mathcal{{M}}(\phi,\mathcal{{F}})$ reduce to $m(\phi,\mathcal{{F}})$, and $\mathcal{{M}}(\phi,p,\mathcal{{F}})$ to $m(\phi,p,\mathcal{{F}})$. Here, without further discussing $\mathcal{{M}}(\varphi,F)$, we immediately define $\mathcal{{M}}(\phi,p,\mathcal{{F}})$ so as not to deviate from our main goal to show that $\mathcal{{M}}(\phi,p,\mathcal{{F}})$ is a class of new double sequences lying between $\mathcal{L}_{p}(\mathcal{{F}})$ and $\mathcal{L}_{\infty} (\mathcal{{F}})$. We then further prove certain conditions under which $\mathcal{{M}}(\phi,p,\mathcal{{F}})$ is same as that of $\mathcal{L}_{p}(\mathcal{{F}})$ and $\mathcal{L}_{\infty} (\mathcal{{F}})$. We can easily see that all results in Section 2 hold for $\mathcal{{M}}(\phi,\mathcal{{F}})$, which is a particular case of $\mathcal{{M}}(\phi,p,\mathcal{{F}})$ with $p = 1$.

## Some interesting results related to $\mathcal{{M}}(\phi,p,\mathcal{{F}})$

### Theorem 3.1

*The sequence space* $\mathcal{{M}}(\phi,p,\mathcal{{F}})$ *is a linear space over* $\mathbb{R}$.

### Proof

Let $x,y \in \mathcal{{M}}(\phi,p,\mathcal{{F}})$ and $\lambda,\mu \in \mathbb{R}$. Then there exists positive numbers $\rho_{1}$ and $\rho_{2}$ such that

$$\sup_{s,t \ge 1}\sup_{\zeta \in \mathcal{{U}}_{st}}\frac{1}{\phi_{st}}\sum _{m,n \in \zeta} \biggl( \mathcal{{F}} \biggl( \frac{ \vert x_{mn} \vert }{\rho_{1}} \biggr) \biggr)^{p} < \infty $$

and

$$\sup_{s,t \ge 1}\sup_{\zeta \in \mathcal{{U}}_{st}}\frac{1}{\phi_{st}}\sum _{m,n \in \zeta} \biggl( \mathcal{{F}} \biggl( \frac{ \vert x_{mn} \vert }{\rho_{2}} \biggr) \biggr)^{p} < \infty. $$

Let $\rho_{3} = \max(2 \vert \lambda \vert \rho_{1},\begin{array}{c} \end{array}2 \vert \mu \vert \rho_{2})$.

(1) $0 < p < 1$. Using the well-known inequality $\vert a + b \vert ^{p} \le \vert a \vert ^{p} + \vert b \vert ^{p}$ for $0 < p < 1$ and the convexity of Orlicz functions, we have

$$\begin{gathered} \sup_{s,t \ge 1}\sup _{\zeta \in \mathcal{{U}}_{st}}\frac{1}{\phi_{st}}\sum_{m,n \in \zeta} \biggl( \mathcal{{F}} \biggl( \frac{ \vert \lambda x_{mn} + \mu y_{mn} \vert }{\rho_{3}} \biggr) \biggr)^{p} \\ \quad \le \sup_{s,t \ge 1}\sup_{\zeta \in \mathcal{{U}}_{st}} \frac{1}{\phi_{st}}\sum_{m,n \in \zeta} \biggl( \mathcal{{F}} \biggl( \frac{ \vert \lambda x_{mn} \vert }{\rho_{3}} \biggr) \biggr)^{p} + \sup _{s,t \ge 1}\sup_{\zeta \in \mathcal{{U}}_{st}}\frac{1}{\phi_{st}}\sum _{m,n \in \zeta} \biggl( \mathcal{{F}} \biggl( \frac{ \vert \mu y_{mn} \vert }{\rho_{3}} \biggr) \biggr)^{p} \\ \quad \le \sup_{s,t \ge 1}\sup_{\zeta \in \mathcal{{U}}_{st}} \frac{1}{\phi_{st}}\sum_{m,n \in \zeta} \biggl( \mathcal{{F}} \biggl( \frac{ \vert \lambda x_{mn} \vert }{\rho_{1}} \biggr) \biggr)^{p} + \sup _{s,t \ge 1}\sup_{\zeta \in \mathcal{{U}}_{st}}\frac{1}{\mathcal{{U}}_{st}}\sum _{m,n \in \zeta} \biggl( \mathcal{{F}} \biggl( \frac{ \vert \mu y_{mn} \vert }{\rho_{2}} \biggr) \biggr)^{p} < \infty, \end{gathered} $$

so that $\lambda x_{mn} + \mu y_{mn} \in \mathcal{{M}}(\phi,p,\mathcal{{F}})$. This proves that $\mathcal{{M}}(\phi,p,\mathcal{{F}})$ is a linear space over $\mathbb{R}$ and so obviously is nonempty.

(2) $1 \le p < + \infty$. It is easy to see that for all $a,b \in \mathbb{R}$, $\vert a + b \vert ^{p} \le 2^{p}( \vert a \vert ^{p} + \vert b \vert ^{p})$ and $\mathcal{{F}}$ is convex, so that, for all $s,t \ge 1$, $\zeta \in \mathcal{{U}}_{st}$,

$$\begin{gathered} \sup_{s,t \ge 1}\sup _{\zeta \in \mathcal{{U}}_{st}}\frac{1}{\phi_{st}}\sum_{m,n \in \zeta} \biggl( \mathcal{{F}} \biggl( \frac{ \vert \lambda x_{mn} + \mu y_{mn} \vert }{\rho_{3}} \biggr) \biggr)^{p} \\ \quad \le \sup_{s,t \ge 1}\sup_{\zeta \in \mathcal{{U}}_{st}} \frac{1}{\phi_{st}}\sum_{m,n \in \zeta} 2^{p} \biggl( \mathcal{{F}} \biggl( \frac{ \vert \lambda x_{mn} \vert }{\rho_{3}} \biggr) \biggr)^{p} + \sup _{s,t \ge 1}\sup_{\zeta \in \mathcal{{U}}_{st}}\frac{1}{\phi_{st}}\sum _{m,n \in \zeta} 2^{p} \biggl( \mathcal{{F}} \biggl( \frac{ \vert \mu y_{mn} \vert }{\rho_{3}} \biggr) \biggr)^{p} \\ \quad \le \sup_{s,t \ge 1}\sup_{\zeta \in \mathcal{{U}}_{st}} \frac{1}{\phi_{st}}\sum_{m,n \in \zeta} \bigl(2^{p} + 1 \bigr) \biggl( \mathcal{{F}} \biggl( \frac{ \vert x_{mn} \vert }{\rho_{1}} \biggr) \biggr)^{p}\\ \qquad {} + \sup_{s,t \ge 1}\sup_{\zeta \in \mathcal{{U}}_{st}} \frac{1}{\phi_{st}}\sum_{m,n \in \zeta} \bigl(2^{p} + 1 \bigr) \biggl( \mathcal{{F}} \biggl( \frac{ \vert y_{mn} \vert }{\rho_{2}} \biggr) \biggr)^{p} \\ \quad < \infty. \end{gathered} $$

This shows that $x,y \in \mathcal{{M}}(\phi,p,\mathcal{{F}}) \Rightarrow \lambda x + \mu y \in \mathcal{{M}}(\phi,p,\mathcal{{F}})$. □

### Remark 3.1

The distance measure between two sequences $x_{n}$ and $y_{n}$ induced by $\operatorname{Ces}_{p}^{q}(\mathcal{{F}})$ can be represented as

$$d_{\operatorname{Ces}_{p}^{q}(\mathcal{{F}})}(x,y) = \Biggl( \sum_{n = 1}^{\infty} \Biggl( \frac{1}{Q_{n}}\sum_{i = 1}^{n} q_{i}f_{i} \bigl( \vert x_{i} - y_{i} \vert \bigr) \Biggr)^{p} \Biggr)^{\frac{1}{p}}. $$

### Theorem 3.2

$\mathcal{{M}}(\phi,p,\mathcal{{F}}) \subseteq \mathcal{{M}}(\psi,p,\mathcal{{F}})$ *if and only if* $\sup_{s,t \ge 1} ( \frac{\phi_{st}}{\psi_{st}} ) < \infty$.

### Proof

Let $x \in \mathcal{{M}}(\phi,p,\mathcal{{F}})$. Then

$$\sup_{s,t \ge 1}\sup_{\zeta \in \mathcal{{U}}_{st}}\frac{1}{\phi_{st}}\sum _{m,n \in \zeta} \biggl( \mathcal{{F}} \biggl( \frac{ \vert x_{mn} \vert }{\rho} \biggr) \biggr)^{p} < \infty\quad \mbox{for some }\rho > 0. $$

Suppose that $\sup_{s,t \ge 1} ( \frac{\phi_{st}}{\psi_{st}} ) < \infty$. Then $\varphi_{st} \le k\psi_{st}$ for some positive number *k* and for all $s,t \in \mathbb{N}$, so $\frac{1}{\psi_{st}} \le \frac{k}{\varphi_{st}}$ for all $s,t \in \mathbb{N}$. Therefore we have

$$\begin{aligned} &\frac{1}{\psi_{st}}\sum_{m,n \in \zeta} \biggl( \mathcal{{F}} \biggl( \frac{ \vert x_{mn} \vert }{\rho} \biggr) \biggr)^{p}\\ &\quad \le \frac{k}{\phi_{st}}\sum _{m,n \in \zeta} \biggl( \mathcal{{F}} \biggl( \frac{ \vert x_{mn} \vert }{\rho} \biggr) \biggr)^{p}\quad \mbox{for each }s,t \in \mathbb{N}\mbox{ and for some }\rho > 0. \end{aligned} $$

Now taking the supremum on both sides we get

$$\begin{aligned} &\sup_{s,t \ge 1}\sup_{\zeta \in \mathcal{{U}}_{st}}\frac{1}{\psi_{st}}\sum _{m,n \in \zeta} \biggl( \mathcal{{F}} \biggl( \frac{ \vert x_{mn} \vert }{\rho} \biggr) \biggr)^{p} \\ &\quad \le k\sup_{s,t \ge 1}\sup _{\zeta \in \mathcal{{U}}_{st}}\frac{1}{\phi_{st}}\sum_{m,n \in \zeta} \biggl( \mathcal{{F}} \biggl( \frac{ \vert x_{mn} \vert }{\rho} \biggr) \biggr)^{p}\quad \mbox{and for some } \rho > 0. \end{aligned} $$

Therefore we have

$$\sup_{s,t \ge 1}\sup_{\zeta \in \mathcal{{U}}_{st}}\frac{1}{\psi_{st}}\sum _{m,n \in \zeta} \biggl( \mathcal{{F}} \biggl( \frac{ \vert x_{mn} \vert }{\rho} \biggr) \biggr)^{p} < \infty\quad \mbox{and for some }\rho > 0. $$

Hence $x \in \mathcal{{M}}(\psi,p,\mathcal{{F}})$.

Conversely, let $\mathcal{{M}}(\phi,p,\mathcal{{F}}) \subseteq \mathcal{{M}}(\psi,p,\mathcal{{F}})$ and suppose that $\sup_{s,t \ge 1} ( \frac{\phi_{st}}{\psi_{st}} ) < \infty$. Then there exist increasing sequences ($s_{i}$) and ($t_{i}$) of natural numbers such that $\lim ( \frac{\phi_{st}}{\psi_{st}} ) = \infty$. Now for every $b \in \mathbb{R}^{ +} $, the set of positive real numbers, there exist $i_{ \circ},j_{ \circ} \in \mathbb{N}$ such that $\frac{\varphi_{s_{i}t_{i}}}{\psi_{s_{i}t_{i}}} > b$ for all $s_{i} \ge i_{ \circ} $ and $t_{i} \ge j_{ \circ} $. Hence $\frac{1}{\psi_{s_{i}t_{i}}} > \frac{b}{\varphi_{s_{i}t_{i}}}$, so that, for some $\rho > 0$,

$$\frac{1}{\psi_{s_{i}t_{i}}}\sum_{m,n \in \zeta} \biggl( \mathcal{{F}} \biggl( \frac{ \vert x_{mn} \vert }{\rho} \biggr) \biggr)^{p} > \frac{b}{\phi_{s_{i}t_{i}}}\sum _{m,n \in \zeta} \biggl( \mathcal{{F}} \biggl( \frac{ \vert x_{mn} \vert }{\rho} \biggr) \biggr)^{p} $$

for all $s_{i} \ge i_{ \circ} $ and $t_{i} \ge j_{ \circ} $. Now taking the supremum over $s_{i} \ge i_{ \circ}$, $t_{i} \ge j_{ \circ} $, and $\zeta \in \mathcal{{U}}_{st}$, we get

2 $$ \sup_{s_{i} \ge i_{ \circ},t_{i} \ge j_{ \circ}} \sup_{\zeta \in \mathcal{{U}}_{st}}\frac{1}{\psi_{s_{i}t_{i}}}\sum _{m,n \in \zeta} \biggl( \mathcal{{F}} \biggl( \frac{ \vert x_{mn} \vert }{\rho} \biggr) \biggr)^{p} > b\sup_{s_{i} \ge i_{ \circ},t_{i} \ge j_{ \circ}} \sup _{\zeta \in \mathcal{{U}}_{st}}\frac{1}{\phi_{s_{i}t_{i}}}\sum_{m,n \in \zeta} \biggl( \mathcal{{F}} \biggl( \frac{ \vert x_{mn} \vert }{\rho} \biggr) \biggr)^{p}. $$

Since (2) holds for all $b \in \mathbb{R}^{ +} $ (we may take *b* sufficiently large), we have

$$\sup_{s_{i} \ge i_{ \circ},t_{i} \ge j_{ \circ}} \sup_{\zeta \in \mathcal{{U}}_{st}}\frac{1}{\psi_{s_{i}t_{i}}}\sum _{m,n \in \zeta} \biggl( \mathcal{{F}} \biggl( \frac{ \vert x_{mn} \vert }{\rho} \biggr) \biggr)^{p} = \infty $$

when $x \in M(\varphi,p,F)$ with $0 < \sup_{s_{i} \ge i_{ \circ},t_{i} \ge j_{ \circ}} \sup_{\zeta \in \mathcal{{U}}_{st}}\frac{1}{\phi_{s_{i}t_{i}}}\sum_{m,n \in \zeta} ( \mathcal{{F}} ( \frac{ \vert x_{mn} \vert }{\rho} ) )^{p} < \infty$.

Therefore $x\notin \mathcal{{M}}(\psi,p,\mathcal{{F}})$. This contradicts to $\mathcal{{M}}(\phi,p,\mathcal{{F}}) \subseteq \mathcal{{M}}(\psi,p,\mathcal{{F}})$. Hence $\sup_{s,t \ge 1} ( \frac{\phi_{st}}{\psi_{st}} ) < \infty$. □

### Corollary 3.1

$\mathcal{{M}}(\phi,p,\mathcal{{F}}) = \mathcal{{M}}(\psi,p,\mathcal{{F}})$ *if and only if* $\sup_{s,t \ge 1}(\eta_{st}) < \infty$ *and* $\sup_{s,t \ge 1}(\eta_{st}^{ - 1}) < \infty$, *where* $\eta_{st} = ( \frac{\phi_{st}}{\psi_{st}} )$ *for all* $s,t \in \mathbb{N}$.

### Corollary 3.2

$\mathcal{{M}}(\phi ) \subseteq \mathcal{{M}}(\phi,p,\mathcal{{F}})$.

### Proof

If $p = 1$ and $\mathcal{{F}}(x) = x$, then $\mathcal{{M}}(\phi ) = \mathcal{{M}}(\phi,p,\mathcal{{F}})$. Also, $\mathcal{{M}}(\phi ) \subseteq \mathcal{{M}}(\phi,p,\mathcal{{F}})$. □

### Theorem 3.3

*The inclusions* $\mathcal{L}_{p}(\mathcal{{F}}) \subseteq \mathcal{{M}}(\phi,p,\mathcal{{F}}) \subseteq \mathcal{L}_{\infty} (\mathcal{{F}})\mathcal{{M}}(\phi,p,\mathcal{{F}})$ *hold*.

### Proof

Let $x \in \mathcal{L}_{p}(\mathcal{{F}})$. Then, for some $\rho > 0$, we have $\sum_{i,j = 1,1}^{\infty,\infty} ( \mathcal{{F}} ( \frac{ \vert x_{mn} \vert }{\rho} ) )^{p} < \infty$. Since ($\varphi_{st}$) is nondecreasing with respect to $s,t \ge 1$, for some $\rho > 0$, we have

$$\begin{aligned} \frac{1}{\phi_{st}}\sum_{m,n \in \zeta} \biggl( \mathcal{{F}} \biggl( \frac{ \vert x_{mn} \vert }{\rho} \biggr) \biggr)^{p} &\le \frac{1}{\phi_{11}}\sum_{m,n \in \zeta} \biggl( \mathcal{{F}} \biggl( \frac{ \vert x_{mn} \vert }{\rho} \biggr) \biggr)^{p} \\ &\le \frac{1}{\phi_{11}}\sum_{i,j = 1,1}^{\infty,\infty} \biggl( \mathcal{{F}} \biggl( \frac{ \vert x_{i,j} \vert }{\rho} \biggr) \biggr)^{p} < \infty. \end{aligned} $$

Hence $\sup_{s,t \ge 1}\sup_{\zeta \in \mathcal{{U}}_{st}}\frac{1}{\phi_{st}}\sum_{m,n \in \zeta} ( \mathcal{{F}} ( \frac{ \vert x_{mn} \vert }{\rho} ) )^{p} < \infty$.

Thus $\mathcal{L}_{p} \subseteq \mathcal{{M}}(\phi,p,\mathcal{{F}})$. Now let $x \in \mathcal{{M}}(\phi,p,\mathcal{{F}})$. Then for some $\rho > 0$, we have

$$\begin{gathered} \sup_{s,t \ge 1}\sup _{\zeta \in \mathcal{{U}}_{st}}\frac{1}{\phi_{st}}\sum_{m,n \in \zeta} \biggl( \mathcal{{F}} \biggl( \frac{ \vert x_{mn} \vert }{\rho} \biggr) \biggr)^{p} < \infty, \\ \sup_{m,n \ge 1}\frac{1}{\phi_{11}}\sum_{m,n \in \zeta} \biggl( \mathcal{{F}} \biggl( \frac{ \vert x_{mn} \vert }{\rho} \biggr) \biggr)^{p} < \infty. \end{gathered} $$

$\Rightarrow \mathcal{{F}} ( \frac{ \vert x_{mn} \vert }{\rho} ) \le ( A\phi_{11} )^{\frac{1}{p}}$ for some $A > 0$ and all $m,n \in \mathbb{N}$. Thus $x \in \mathcal{L}_{\infty} (\mathcal{{F}})$. □

### Theorem 3.4

*Let* $\mathcal{{F}}$, $\mathcal{{F}}_{1}$, $\mathcal{{F}}_{2}$ *be Orlicz functions satisfying* $\Delta_{2}$-*condition*. *Then*

1. $\mathcal{{M}}(\phi,p,\mathcal{{F}}_{1}) \subseteq \mathcal{{M}}(\phi,p,\mathcal{{F}} \circ \mathcal{{F}}_{1})$,
2. $\mathcal{{M}}(\phi,p,\mathcal{{F}}_{1}) \cap \mathcal{{M}}(\phi,p,\mathcal{{F}}_{2}) = \mathcal{{M}}(\phi,p,\mathcal{{F}}_{1} + \mathcal{{F}}_{2})$.

### Proof

(1) Let $x \in \mathcal{{M}}(\phi,p,\mathcal{{F}}_{1})$. Then there exists $\rho > 0$ such that

$$\sup_{s,t \ge 1}\sup_{\zeta \in \mathcal{{U}}_{st}}\frac{1}{\phi_{st}}\sum _{m,n \in \zeta} \biggl( \mathcal{{F}} \biggl( \frac{ \vert x_{mn} \vert }{\rho} \biggr) \biggr)^{p} < \infty. $$

Let $0 < \varepsilon < 1$ and *δ* with $0 < \delta < 1$ be such that $F(t) < \varepsilon$, $0 < t \le \delta$. Put $t_{mn} = \mathcal{{F}}_{1} ( \frac{ \vert x_{mn} \vert }{\rho} )$ and for any $\zeta \in \mathcal{{U}}_{s}$, consider

$$\sum_{m,n \in \zeta} \bigl( \mathcal{{F}}(t_{mn}) \bigr)^{p} = \sum_{1} \bigl( \mathcal{{F}}(t_{mn}) \bigr)^{p} + \sum _{2} \bigl( \mathcal{{F}}(t_{mn}) \bigr)^{p}, $$

where the first sum is over $t_{mn} \le \delta$, and the second is over $t_{mn} > \delta$. From the remark we have

3 $$ \sum_{1} \bigl( \mathcal{{F}}(t_{mn}) \bigr)^{p} \le \bigl( \mathcal{{F}}(1) \bigr)^{p}\sum _{1} \bigl( t_{mn}^{p} \bigr) \le \bigl( \mathcal{{F}}(2) \bigr)^{p}\sum_{1} \bigl( t_{mn}^{p} \bigr), $$

and for $t_{mn} > \delta$, we use the fact that

$$t_{mn} < \frac{t_{mn}}{\delta} < 1 + \frac{t_{mn}}{\delta}. $$

Since $\mathcal{{F}}$ is nondecreasing and convex, we have

$$\mathcal{{F}}(t_{mn}) \le \mathcal{{F}} \biggl( 1 + \frac{t_{mn}}{\delta} \biggr) < \frac{1}{2}\mathcal{{F}}(2) + \frac{1}{2}\mathcal{{F}} \biggl( \frac{2t_{mn}}{\delta} \biggr). $$

Since $\mathcal{{F}}$satisfies $\Delta_{2}$-condition, we have

$$\mathcal{{F}}(t_{mn}) < \frac{1}{2}k\frac{t_{mn}}{\delta} \mathcal{{F}}(2) + \frac{1}{2}k\frac{t_{mn}}{\delta} \mathcal{{F}}(2) = k \frac{t_{mn}}{\delta} \mathcal{{F}}(2). $$

Hence

$$\bigl( \mathcal{{F}}(t_{mn}) \bigr)^{p} < \biggl( k\frac{t_{mn}}{\delta} \mathcal{{F}}(2) \biggr)^{p}. $$

Therefore

4 $$ \sum_{2} \bigl( \mathcal{{F}}(t_{mn}) \bigr) \le \max \biggl( 1, \biggl( \frac{k\mathcal{{F}}(2)}{\delta} \biggr)^{p} \biggr)^{p}\sum_{m,n \in \zeta} \bigl( t_{mn}^{p} \bigr). $$

By (3) and (4) we have $\mathcal{{M}}(\phi,p,\mathcal{{F}}_{1}) \subseteq \mathcal{{M}}(\phi,p,\mathcal{{F}} \circ \mathcal{{F}}_{1})$.

(2) The proof follows from the inequality

$$\begin{gathered} \sup_{s,t \ge 1}\sup _{\zeta \in \mathcal{{U}}_{st}}\frac{1}{\phi_{st}} \biggl\{ \sum _{m,n \in \zeta} \biggl( (\mathcal{{F}}_{1} + \mathcal{{F}}_{2}) \biggl( \frac{ \vert x_{mn} \vert }{\rho} \biggr) \biggr)^{p} \biggr\} ^{\frac{1}{p}} \\ \quad \le \sup_{s,t \ge 1}\sup_{\zeta \in \mathcal{{U}}_{st}} \frac{1}{\phi_{st}} \biggl\{ \sum_{m,n \in \zeta} \biggl( \mathcal{{F}}_{1} \biggl( \frac{ \vert x_{mn} \vert }{\rho} \biggr) \biggr)^{p} \biggr\} ^{\frac{1}{p}} + \sup_{s,t \ge 1}\sup _{\zeta \in \mathcal{{U}}_{st}}\frac{1}{\phi_{st}} \biggl\{ \sum _{m,n \in \zeta} \biggl( \mathcal{{F}}_{2} \biggl( \frac{ \vert x_{mn} \vert }{\rho} \biggr) \biggr)^{p} \biggr\} ^{\frac{1}{p}}\\ \quad < \infty. \end{gathered} $$

 □

### Theorem 3.5

*The* $\mathcal{{M}} ( \phi,p,\mathcal{{F}} )$ *satisfy the following relations*:

1. $\mathcal{{M}} ( \phi,p,\mathcal{{F}} ) = \mathcal{L}_{p}(\mathcal{{F}})$ *if and only if* $\sup_{s,t \ge 1}(\varphi_{st}) < \infty$,
2. $\mathcal{{M}} ( \phi,p,\mathcal{{F}} ) = \mathcal{L}_{\infty} (\mathcal{{F}})$ *if and only if* $\sup_{s,t \ge 1} ( \frac{st}{\phi_{st}} ) < \infty$.

### Proof

(1) If we take $\varphi_{st} = 1$ for all $s,t \in \mathbb{N}$, then we have $\mathcal{{M}} ( \phi,p,\mathcal{{F}} ) = \mathcal{L}_{p}(\mathcal{{F}})$.

(2) By Theorem 3.3 we easily get that $\mathcal{{M}} ( \phi,p,\mathcal{{F}} ) = \mathcal{L}_{\infty} (\mathcal{{F}})$ if and only if $\sup_{s,t \ge 1} ( \frac{st}{\phi_{st}} ) < \infty$. □

### *k*-means algorithm for $\mathcal{{M}} ( \phi,p,\mathcal{{F}} )$ distance measure

Let $X = \{ x_{1},x_{2},\ldots,x_{n} \}$ be a given data set. Then the proposed clustering algoritm works as follows.

1. Select first *k* data points as the cluster center $x_{k} = \{ x_{1},x_{2},\ldots,x_{k} \}$ (where *k* is the number of clusters).
2. Compute the distance between each data point and cluster center through $\mathcal{{M}} ( \phi,p,\mathcal{{F}} )$ distance measure.
3. Put the data point into that cluster whose $\mathcal{{M}} ( \phi,p,\mathcal{{F}} )$ distance with its center is minimal.
4. Redefine cluster centers for newly evolved clusters due to the above steps; the new cluster centers are computed as $c_{i} = \frac{1}{k_{i}}\sum_{j = 1}^{k_{i}} x_{i}$, where $k_{i}$ is the number of points in the *i*th cluster.
5. Repeat Step 1 to Step 4 until the difference between two consecutive cluster centers becomes less than a desired small number.

### Clustering by using the induced $\mathcal{{M}} ( \phi,p,\mathcal{{F}} )$ distance measure

**Figure 2:**
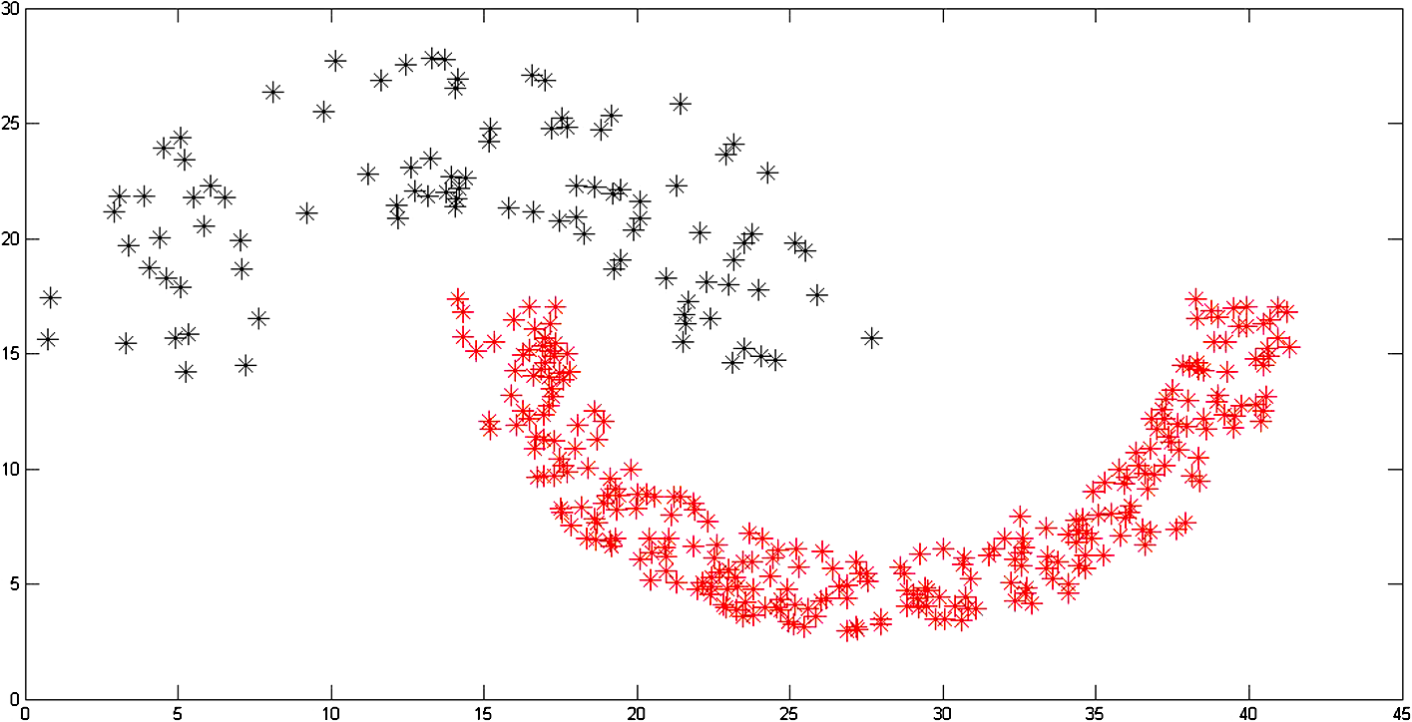
**Two-moon data set.**

**Figure 3:**
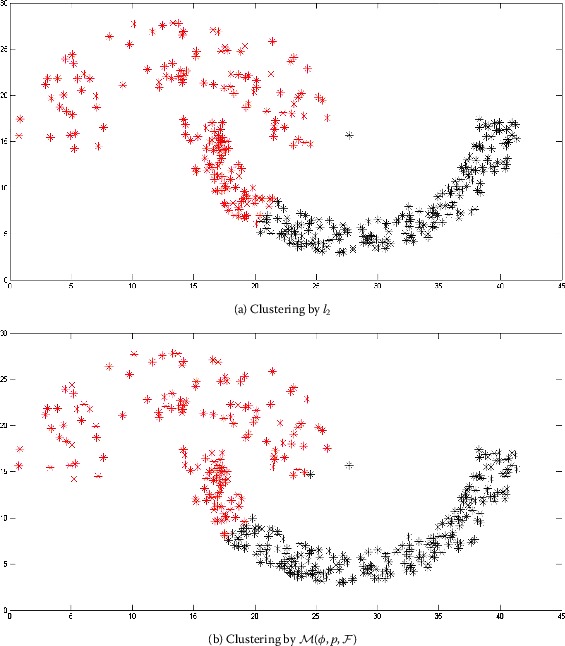
**Obtained clustering results for two-moon data set.**

**Figure 4:**
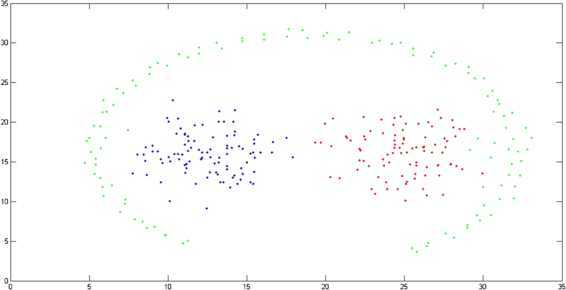
**Path-based data set.**

**Figure 5:**
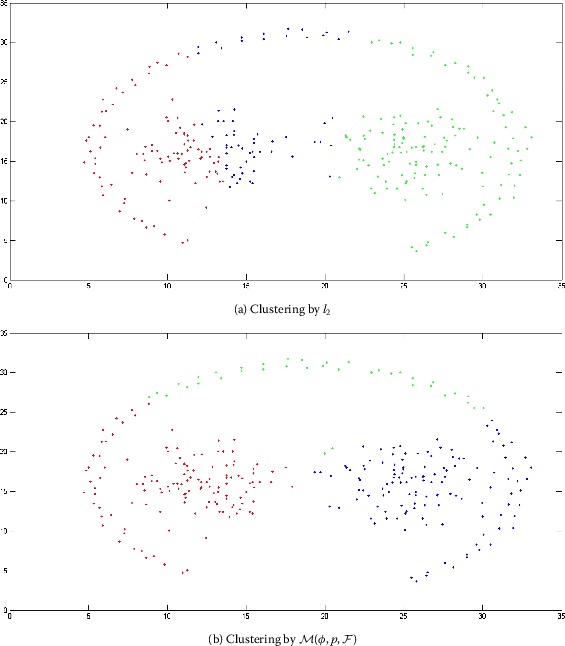
**Obtained clustering results for two-moon data set.**

Two-moon and path-based data sets are artificially designed as nonconvex collections of points [36, 37]. The original shapes of the two-moon and path-based data are represented in Figures 2 and 4, respectively. The clustering on these two data sets is carried out by the algorithm dissussed in Section 3.1. In the case of a two-moon data set, for making simulation process simple, we take $\varphi = 1$, $\forall m,n$, $p = 1$, and $F(x) = \vert x \vert $. In Figure 3(a), it is shown that the clustering accuracy of the *k*-means clustering algorithm is 78% over the two-moon data set, whereas the clustering accuracy of our modefied algorithm *k*-means clustering is 84% (Figure 3(b)). Moreover, in the case of a path-based data set, we take $\varphi = n$, $\forall m,n$, $p = 1$, and $F(x) = \vert x \vert $. The clustering accuracy of the path-based data set by using the *k*-mean clustering algorithm is 45%, whereas by using the proposed modefied *k*-means clustering algorithm it is 67% as shown in Figures 5(a) and 5(b), respectively.

## Conclusions

The parameters *ϕ*, *p*, $\mathcal{{F}}$ involved in the sequence space $\mathcal{{M}} ( \phi,p,\mathcal{{F}} )$ give additional three degrees of freedom to its induced distance measure. Therefore, it is more flexible in comparison to the $l_{p}$ or weighted $l_{p}$ distance measure. The flexibility in the distance measure can be judiciously used in the clustering of the real-world data sets. We have proposed only a modified *k*-means clustering algorithm; in the similar fashion, other distance-based clustering algorithms can also be modified. So, improvement in many clustering algorithms is possible due to a distance measure of $\mathcal{{M}} ( \phi,p,\mathcal{{F}} )$. We have shown the efficacy of an $\mathcal{{M}} ( \phi,p,\mathcal{{F}} )$-based *k*-means clustering algorithm over the $l_{2}$-based *k*-means clustering algorithm on the basis of better clustering accuracy obtained for a two-moon data set and path-based data set.
